# Phase 1 dose-escalation study of the PARP inhibitor CEP-9722 as monotherapy or in combination with temozolomide in patients with solid tumors

**DOI:** 10.1007/s00280-014-2486-9

**Published:** 2014-06-01

**Authors:** Ruth Plummer, Peter Stephens, Louiza Aissat-Daudigny, Anne Cambois, Gilbert Moachon, Peter D. Brown, Mario Campone

**Affiliations:** 1 Northern Centre for Cancer Care, Newcastle upon Tyne, NE7 7DN UK; 2 Teva Laboratories France, 110 Esplanade du Général de Gaulle, 92931 La Défense Cedex, France; 3 Formerly of Cephalon France, 20 Rue Charles Martigny, 94700 Maisons-Alfort, France; 4 Teva Branded Pharmaceutical Products R&D, Inc., 41 Moores Rd, Frazer, PA 19355 USA; 5 Institut de Cancérologie de l’Ouest-René Gauducheau, Boulevard Jacques Monod, 44805 Saint Herblain-Nantes Cedex, France

**Keywords:** PARP inhibitor, Solid tumors, Phase 1, Dose escalation, Temozolomide, Maximum tolerated dose

## Abstract

**Purpose:**

Poly(ADP-ribose) polymerase-1 (PARP-1) is a nuclear enzyme important in DNA repair. PARP-1 activation at points of DNA strand break results in poly(ADP-ribose) polymer formation, opening the DNA structure, and allowing access of other repair enzymes. CEP-9722 inhibits PARP-1 and PARP-2 and is designed to potentiate DNA-damaging chemotherapies.

**Methods:**

This dose-escalating phase 1 study assessed the safety, maximum tolerated dose (MTD), and pharmacokinetics/pharmacodynamics of CEP-9722 plus temozolomide in adults with solid tumors. Tumor response was also assessed. Participants received a 14-day cycle of CEP-9722 (days 1 and 3–5 or days 1–5), followed by 28-day cycles of CEP-9722 plus temozolomide 150 mg/m^2^ on days 1–5. The initial CEP-9722 dose (cohort 1) was 150 mg/day; dose escalation followed a modified Fibonnaci sequence.

**Results:**

Twenty-six patients received CEP-9722 150–1,000 mg/day combined with temozolomide. Dose-limiting toxicities of asthenia and persistent weight loss at 1,000 mg/day resulted in 750 mg/day being defined as the MTD and recommended dose for further study. Overall, 24 (92 %) patients had treatment-related adverse events (TRAEs), mostly grade 1 or 2, with nausea, vomiting, and diarrhea having the strongest relation to CEP-9722. Four patients had grade 3 TRAEs (asthenia, myositis, diarrhea, and fatigue). Systemic exposure generally increased with dosage, with high inter- and intra-patient variability at all doses. Pharmacodynamic assessment confirmed PARP inhibition although no dose response was apparent. One patient with melanoma achieved a partial response (1,000 mg/day).

**Conclusions:**

CEP-9722 was adequately tolerated with temozolomide; the MTD was 750 mg/day. Only limited clinical activity was observed.

**Electronic supplementary material:**

The online version of this article (doi:10.1007/s00280-014-2486-9) contains supplementary material, which is available to authorized users.

## Introduction

Poly(ADP-ribose) polymerases (PARPs) are a family of highly conserved enzymes involved in the regulation of numerous cellular functions [1, 2]. PARP-1 and PARP-2 are nuclear enzymes that are activated in response to DNA damage [3, 4]. Once PARP-1 is activated, it forms long branched chains of poly(ADP-ribose) (PAR) on DNA-associated proteins [5]. This creates a negatively charged environment and allows access for the enzymes responsible for base excision repair. The auto-ADP ribosylation of PARP-1 results in its release from DNA. PARP-1 is also involved in other DNA repair processes such as pathways of double-strand break repair [6].

Given that many anticancer therapies act by damaging the DNA in tumor cells, investigating the therapeutic potential of agents that block PARP-1, and therefore DNA repair, has generated much interest [7, 8]. PARP inhibitors have been combined in clinical trials with chemotherapies such as temozolomide [9] and topotecan [10–12]. The therapeutic potential of PARP inhibitors has also been studied in patients with tumors that have defects in the DNA repair process such as BRCA loss of function mutations. In these patients, the tumor cells have an impaired homologous recombination DNA repair process and are particularly vulnerable to further reductions in DNA repair capacity by monotherapy treatment with PARP inhibitors [13, 14].

CEP-9722 is a pro-drug of CEP-8983, a potent inhibitor of PARP-1 and PARP-2 with enzyme IC_50_ values of 20 and 6 nm, respectively. The CEP-9722 pro-drug was developed in order to try to overcome the low oral bioavailability of the poorly soluble CEP-8983. In nonclinical studies, CEP-8983 increased the sensitivity of chemoresistant tumor cells to temozolomide, both in vitro and in xenograft models. CEP-8983 was also shown to cause only modest increases in the myelotoxicity of temozolomide in studies using the CFU-GM colony-forming assay with human bone marrow cells [15]. This last finding was considered important since the potentiation of myelosuppression by other PARP inhibitors has been shown to limit the ability to co-administer these agents with full-dose chemotherapy [12, 16].

The purpose of this first-in-human study was to evaluate the maximum tolerated dose (MTD), safety, pharmacokinetics, pharmacodynamics, and efficacy of the oral PARP-1/-2 inhibitor CEP-9722 in combination with oral temozolomide in patients with advanced solid tumors.

## Methods

### Study design

This was an open-label, nonrandomized, multicenter, dose-escalating phase 1 clinical trial. Patients were enrolled at 1 center in France and 1 center in the United Kingdom between May 19, 2009, and June 20, 2011. The primary objective was to identify the MTD of CEP-9722 in combination with temozolomide. Secondary objectives were to determine the dose range of CEP-9722 that resulted in PARP inhibition in peripheral blood mononuclear cells (PBMCs), to characterize the pharmacokinetics of CEP-8983, and to document any antitumor activity. Dose escalation approximated to a modified Fibonacci sequence and followed a standard 3 + 3 design.

The study consisted of a screening period to determine patient eligibility, a 14-day cycle in which CEP-9722 was administered once daily as monotherapy, and subsequent 28-day cycles in which CEP-9722 and temozolomide were administered as combination therapy. In cycle 1, the first patient in each dosing cohort received CEP-9722 on days 1, 3, 4, and 5, while other patients in each cohort received CEP-9722 on days 1–5. In cycle 2, patients received CEP-9722 and temozolomide on days 1–5 (Supplemental Fig.). CEP-9722 was escalated from a starting dose of 150 mg/day up to 1,000 mg/day, and temozolomide was given at the dose of 150 mg/m^2^/day at all dose levels of CEP-9722. Both CEP-9722 and temozolomide were administered in the fasted state.

Patients were enrolled in cohorts of 3. Enrollment in the next cohort commenced when the third patient in the current cohort had completed day 28 of cycle 2 without a dose-limiting toxicity (DLT). Patients were considered evaluable for determination of the MTD provided that they had received all doses of study treatment in cycles 1 and 2. If a DLT was observed in cycle 1 or 2 in 1 patient, 3 additional patients were treated at this dose level. If only 1 DLT was observed in the cohort of 6 patients, the subsequent cohort was opened for enrollment; however, if a DLT was observed in ≥2 patients in a cohort of 3 or 6 patients, dose escalation was stopped and that dose was defined as being above the dose to be recommended for further study.

DLT was defined as any ≥grade 3 nonhematologic toxicity, with the exception of alopecia, vomiting, or nausea in the absence of effective antiemetic treatment, or diarrhea in the absence of effective antidiarrheal treatment; grade 4 neutropenia persisting for more than 7 days or grade 3 neutropenia with fever of 38.5 °C (101.3 °F) or higher; grade 4 thrombocytopenia or grade 3 thrombocytopenia with clinically significant bleeding; QTc interval >500 ms or QTc variation from baseline >60 ms on ≥2 electrocardiograms (ECGs) performed at the same visit; persistence of ≥grade 2 nonhematologic toxicity at the initiation of cycles 2 or 3; or any prolonged ≥grade 2 myelotoxicity that delays initiation of cycle 3 by more than 1 week.

Patients withdrawing from the study for any reason other than a DLT before completing cycle 2 were replaced. Patients demonstrating clinical benefit or evidence of tumor response and/or no significant toxicity were eligible to receive additional cycles of CEP-9722 in combination with temozolomide per investigator discretion.

### Patient population

Men and women aged ≥18 years with a histologically or cytologically confirmed advanced solid tumor considered unresponsive or poorly responsive to accepted therapies were eligible for study enrollment. Other key inclusion criteria were adequate renal and hepatic function, and bone marrow reserve; an Eastern Cooperative Oncology Group (ECOG) performance status of 0–2; a life expectancy of ≥12 weeks; evaluable disease by Response Evaluation Criteria in Solid Tumors (RECIST) 1.0 criteria; no prior chemotherapy within past 3 weeks, nitrosourea treatment within past 6 weeks, or radiotherapy within past 4 weeks; written informed consent; and agreement to use appropriate contraceptive measures.

The main exclusion criteria were a primary brain tumor requiring a systemic premedication with anticonvulsive agents; brain metastases with symptoms in the prior 4 weeks; marked baseline prolongation of QT/QTc interval, risk factors for torsade de pointes, family history of long QT syndrome, heart failure, or use of concomitant treatment known to prolong QT/QTc interval; previous hypersensitivity reactions to any of the components of CEP-9722, temozolomide, or dacarbazine; an active gastroduodenal ulcer, uncontrolled high blood pressure (systolic >150 mmHg and diastolic >90 mmHg with medication), uncontrolled diabetes mellitus, uncontrolled angina pectoris, recent myocardial infarction, cerebrovascular event within 6 months of study entry, or preexisting coagulopathy; concomitant uncontrolled infection; or severe systemic disease.

The study was conducted in full accordance with the Good Clinical Practice: Consolidated Guidance approved by the International Conference on Harmonisation and applicable national and local laws and regulations.

### Safety and efficacy assessments

Safety was assessed using adverse event data, clinical laboratory test results, vital signs measurement, ECG findings, physical examination findings, ECOG performance status, and concomitant medication usage.

Tumor assessment was performed within 4 weeks before the first dose of study drug and within the fourth week of cycle 2 using computed tomography or magnetic resonance imaging with contrast. In patients who continued treatment, tumor assessment was performed every 2 cycles and at the end of treatment visit, at the discretion of the investigator.

### Pharmacokinetic/pharmacodynamic assessments

Pharmacokinetic serum samples were collected on day 1 of cycle 1 pre-dose and 0.5, 1, 2, 3, 4, 6, 8, 10, and 24 h post-dose; and on day 5 of cycle 1 and cycle 2 pre-dose, and 0.5, 1, 2, 3, 4, 6, 8, 10, 24, and 48 h post-dose. Urine samples were obtained on day 1 of cycle 1 during 4 intervals in the 24 h following study drug administration. Pharmacokinetic parameters (area under the plasma drug concentration–time curve from time 0 to infinity [AUC_0–∞_], AUC from time 0 to the time of the last measurable drug concentration [AUC_0–t_], AUC for 1 dosing interval [AUC_τ_] following multiple doses, maximum observed plasma concentration [*C*_max_], and time to maximum observed plasma concentration [*T*_max_]) were summarized at each visit (day 1 of cycle 1 and day 5 of cycles 1 and 2) using descriptive statistics.

Pharmacodynamics were assessed by the measurement of PAR concentrations in PBMCs. PBMCs were isolated at site, and the frozen cell pellet was sent to a central laboratory (Trevigen Inc., Gaithersburg, MD, USA) for processing and analysis by PAR enzyme-linked immunosorbent assay (ELISA) [17]. Pharmacodynamic samples were collected on 2 consecutive days at screening visits and on days 1, 5 (pre-dose and 2 h [cycle 1 only], 6 h, and 24 h post-dose), and 8 (at the time of hematology laboratory testing). The PAR concentrations in the 2 screening samples and the day 1 pre-dose sample were averaged to provide a baseline value, and the percentage inhibition from baseline was calculated in the post-dose samples.

## Results

### Patient disposition and demographics

In total, 33 patients with solid tumors were screened for study eligibility; of these, 26 patients met the entry criteria and were enrolled. The mean age of the patients was 52.8 (range 18–71) years. All of the patients had had previous chemotherapy; 23 patients (88 %), surgery; 13 patients (50 %), radiotherapy; and 10 patients (38 %), target therapy (Table 1).

**Table 1:** Patient demographics and disease characteristics

*N* patients treated (%)	CEP-9722 dose level (mg/day)					Total
	150 3	300 6	500 3	750 11	1,000 3	26
Age in years, mean ± SD	55.3 ± 11.72	53.7 ± 12.91	52.3 ± 7.23	50.6 ± 15.10	57.0 ± 7.94	52.8 ± 12.24
Gender						
Female	3 (100)	4 (67)	2 (67)	7 (64)	2 (67)	18 (69)
Male	0	2 (33)	1 (33)	4 (36)	1 (33)	8 (31)
Smoking status/medications affecting gastric pH						
Smoker	0	4 (67)	0	2 (18)	1 (33)	7 (27)
Nonsmoker	3 (100)	2 (33)	3 (100)	9 (82)	2 (67)	19 (73)
Primary cancer						
Breast Ovarian	2 (67) 0	1 (17) 1 (17)	0 0	4 (36) 2 (18)	0 1 (33)	7 (27) 4 (15)
Colorectal	0	0	1 (33)	1 (9)	0	2 (8)
Other^a^	1 (33)	4 (67)	2 (67)	4 (36)	2 (67)	13 (50)
ECOG status						
0	2 (67)	2 (33)	2 (67)	4 (36)	1 (33)	11 (42)
1	1 (33)	4 (67)	1 (33)	7 (64)	1 (33)	14 (54)
2	0	0	0	0	1 (33)	1 (4)
Prior anticancer radiotherapy	3 (100)	2 (33)	1 (33)	7 (64)	0	13 (50)
Prior systemic anticancer chemotherapy	3 (100)	6 (100)	3 (100)	11 (100)	3 (100)	26 (100)

*ECOG* Eastern Cooperative Oncology Group, *SD* standard deviation

^a^Other includes 1 each: abdominal/pelvic sarcoma, adenocarcinoma of the jejunum, atypical carcinoid cancer, cervix uteri cancer, cholangiocarcinoma, external ear cancer, gallbladder cancer, esophageal cancer, lung cancer, melanoma, prostate cancer, stomach cancer, and urothelial carcinoma

### Maximum tolerated dose

Three patients were enrolled at the starting dose of 150 mg/day and completed the treatment in the first 2 cycles without DLT. One patient in the second cohort (300 mg/day) had a DLT of grade 3 fatigue, and the cohort was expanded to six patients. No other DLTs were observed in the second or third cohort (500 mg/day, 3 patients). One patient in the fourth cohort (750 mg/day) had a grade 3 myositis on day 15 of cycle 1, and the cohort was expanded to six patients. No other DLTs were observed, and three patients were enrolled in the fifth cohort (1,000 mg/day). Two of these patients developed DLTs (grade 3 asthenia on day 5 of cycle 1; persistent grade 2 weight loss in cycle 2). It was therefore concluded that, at a dose of 1,000 mg/day, the tolerable dose had been exceeded, and 3 additional patients were enrolled at a dose of 750 mg/day without DLT.

In total, 8 patients (31 %) withdrew from the study before completion of day 28 of cycle 2. The recommended phase 2 dose for CEP-9722 was established at a dose of 750 mg/day, days 1–5, in combination with temozolomide at a dose of 150 mg/m^2^/day, days 1–5, in a 28-day cycle.

### Safety

All patients enrolled in the study had 1 or more adverse events during the treatment period. The most commonly occurring adverse events (≥20 % of patients) were nausea (77 %), vomiting (65 %), constipation (50 %), headache (50 %), diarrhea (42 %), asthenia (42 %), abdominal pain (35 %), anorexia (35 %), fatigue (31 %), and anemia (23 %).

The most common severe adverse events were abdominal pain (1 patient at 300 mg/day, 2 patients at 750 mg/day), asthenia (1 patient each at 300 and 1,000 mg/day), and noncardiac chest pain (2 patients at 750 mg/day). Six patients had grade 3–4 laboratory hematologic toxicities (5 lymphopenia [3 at 750 mg/day and 1 each at 300 and 1,000 mg/day] and 1 anemia at 750 mg/day).

Dose delays due to hematological toxicity occurred in three patients (all at a CEP-9722 dose of 750 mg/day). Four patients withdrew due to adverse events: 2 due to asthenia (750 and 1,000 mg/day; both possibly/probably related to treatment), 1 due to weight loss (1,000 mg/day, possibly related), and 1 due to respiratory distress (150 mg/day, not considered related). The patient with respiratory distress died. There were four additional deaths, all due to disease progression, which were not considered treatment related.

Treatment-related adverse events during cycle 1 (monotherapy) and subsequent cycles (combination therapy) are presented in Table 2. Events of headache, diarrhea, nausea, and vomiting showed the clearest relationship to dose during monotherapy. Similar trends were observed during combination therapy (cycles 2 and beyond). Grade 3 TRAEs occurred in two patients during monotherapy with CEP-9722 (asthenia and myositis), and in two patients during combination therapy (diarrhea and fatigue).

**Table 2:** All grades of treatment-related adverse events in at least two patients who received CEP-9722 alone or combined with temolozomide

*N* patients treated (%)	CEP-9722 dose level (mg/day)					Total
	150 3	300 6	500 3	750 11	1,000 3	26
Cycle 1: single-agent CEP-9722						
Nausea	0	1 (17)	1 (33)	8 (73)	2 (67)	12 (46)
Diarrhea	0	0	0	5 (45)	2 (67)	7 (27)
Vomiting	0	1 (17)	0	4 (36)	2 (67)	7 (27)
Abdominal pain	0	0	0	2 (18)	0	2 (8)
Constipation	0	0	0	2 (18)	0	2 (8)
Asthenia	0	1 (17)	0	3 (27)	1 (33)	5 (19)
Fatigue	1 (33)	1 (17)	0	2 (18)	0	4 (15)
Blood creatinine phosphokinase increased	0	0	0	1 (9)	1 (33)	2 (8)
Anorexia	0	0	0	1 (9)	2 (67)	3 (12)
Headache	0	1 (17)	2 (67)	5 (45)	2 (67)	10 (38)
Cycles ≥ 2: CEP-9722 combined with temozolomide						
Leukopenia	1 (33)	0	0	2 (18)	0	3 (12)
Anemia	0	0	1 (33)	1 (9)	0	2 (8)
Neutropenia	1 (33)	0	0	1 (9)	0	2 (8)
Thrombocytopenia	1 (33)	0	0	1 (9)	0	2 (8)
Nausea	1 (33)	3 (50)	1 (33)	8 (73)	2 (67)	15 (58)
Vomiting	1 (33)	1 (17)	1 (33)	8 (73)	2 (67)	13 (50)
Diarrhea	0	0	0	3 (27)	2 (67)	5 (19)
Constipation	0	0	0	2 (18)	1 (33)	3 (12)
Dyspepsia	0	0	0	2 (18)	0	2 (8)
Asthenia	0	0	2 (67)	4 (36)	0	6 (23)
Fatigue	0	1 (17)	0	4 (36)	1	6 (23)
Gamma-glutamyltransferase increased	0	0	1 (33)	2 (18)	0	3 (12)
Hemoglobin decreased	0	1 (17)	0	2 (18)	0	3 (12)
Weight decreased	0	1 (17)	0	1 (9)	1 (33)	3 (12)
Aspartate aminotransferase increased	0	0	1 (33)	1 (9)	0	2 (8)
Anorexia	0	1 (17)	0	1 (9)	2 (67)	4 (15)
Headache	0	2 (33)	2 (67)	5 (45)	2 (67)	11 (42)

Central review of ECGs showed no clinically significant abnormalities.

### Efficacy

In total, 22 of the 26 patients enrolled in the study were evaluated for efficacy (Table 3). One patient demonstrated a confirmed partial response according to RECIST criteria, four patients had stable disease, and 17 patients had progressive disease. The patient demonstrating a partial response (a 58 % reduction in target lesions, as defined by RECIST criteria) was a 66-year-old man with melanoma previously treated with paclitaxel and pazopanib who received CEP-9722 1,000 mg/day. The partial response was first observed after 6 cycles of treatment and lasted 5.8 months. The patient did not experience disease progression during the study; however, CEP-9722 and temozolomide were stopped after cycle 12 due to concerns over temozolomide cumulative toxicity.

**Table 3:** Best tumor response according to RECIST 1.0 criteria

Cohort	CEP-9722 dosage (mg/day)	Patients evaluated	Partial response	Stable disease	Progressive disease
1	150	2	0	0	2
2	300	6	0	1	5
3	500	3	0	2	1
4	750	9	0	1	8
5	1,000	2	1	0	1
Total		22	1	4	17

*RECIST* response evaluation criteria in solid tumors

### Pharmacokinetics

**Fig. 1:**
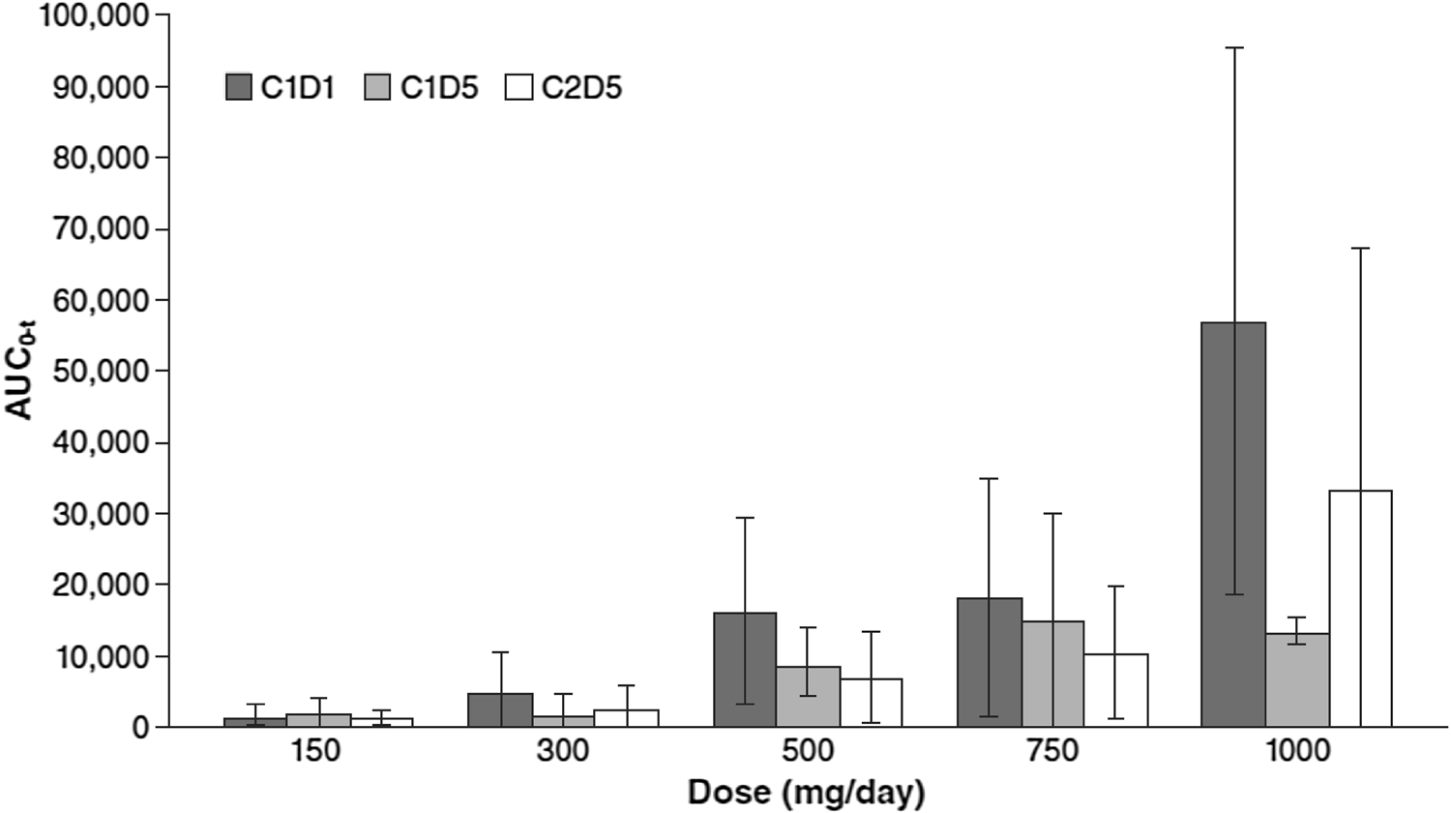
Mean (SD) area under the plasma drug concentration–time curve from time 0 to the time of the last measurable drug concentration (AUC_0–t_) of CEP-8983 after CEP-9722 administration on days 1 and 5 of cycle 1 (C1D1, C1D5) and on day 5 of cycle 2 (C2D5)

Conversion of CEP-9722 into its active form is rapid; thus, systemic exposure was assessed by measuring the plasma concentration of CEP-8983. In general, systemic exposure increased with dose, but there was a high degree of inter- and intra-patient variability (Fig. 1). Smoking and the concomitant use of agents that raise gastric pH were associated with low plasma exposure (Table 4). In addition, a general trend toward higher exposure on day 1 of cycle 1 than that on day 5 of cycle 1 and day 5 of cycle 2 was observed, which may be related to changes in drug absorption or metabolism. Mean AUC_0–t_ of CEP-8983 for nonsmokers not receiving gastric pH-modifying medications versus those in smokers and/or patients receiving gastric pH-modifying medications were, respectively, 4,960.6 ng h/mL versus 2,747.3 ng h/mL for day 1 of cycle 1; 3,138.0 ng h/mL versus 1,339.3 ng h/mL for day 5 of cycle 1; and 2,574.4 ng h/mL versus 1,457.9 ng h/mL for day 5 of cycle 2.

**Table 4:** Pharmacokinetic parameters for CEP-8983 by smoking status/receiving medications that affect gastric pH (day 5 of cycle 1)

Pharmacokinetic parameters, mean ± SD	Nonsmoker without medication affecting gastric pH *n* = 13	Smoker and/or with medication affecting gastric pH *n* = 11
*C*_max_ (ng/mL)	607.5 ± 319.87	352.5 ± 380.57
AUC_0–∞_ (ng h/mL)	4,111.0 ± 4,126.79	2,838.9 ± 5,740.57
AUC_0–*t*_ (ng h/mL)	3,138.0 ± 2,160.74	1,339.3 ± 2,127.07
AUC_τ_ (ng h/mL)	3,851.1 ± 3,578.78	2,304.7 ± 4,161.29
*t*_max_ (h)	0.6 ± 0.66	0.6 ± 0.66
*t*_1/2_ (h)	1.0 ± 0.59	0.7 ± 0.77
CL/F (L/h)	20.4 ± 25.80	67.1 ± 62.14
V/F(L)	58.1 ± 60.23	102.3 ± 100.76
Percentage extrapolation (%)	3.7 ± 3.82	5.5 ± 8.29
*λ*_z_ (1/h)	0.1 ± 0.13	0.2 ± 0.21

*AUC*_*0*–*∞*_ area under the plasma drug concentration–time curve from time 0 to infinity, *AUC*_*0*–*t*_ area under the plasma drug concentration–time curve from time 0 to the time of the last measurable drug concentration, *AUC*_*τ*_ area under the plasma drug concentration–time curve for 1 dosing interval, *CL/F* total body clearance from plasma after oral administration, *C*_max_ maximum observed plasma concentration, *λ*_*z*_ terminal plasma elimination rate-constant, *t*_*1/2*_ half-life, *t*_max_ time to maximum observed plasma concentration, *V/F* apparent volume of distribution after oral administration

### Pharmacodynamics

**Fig. 2:**
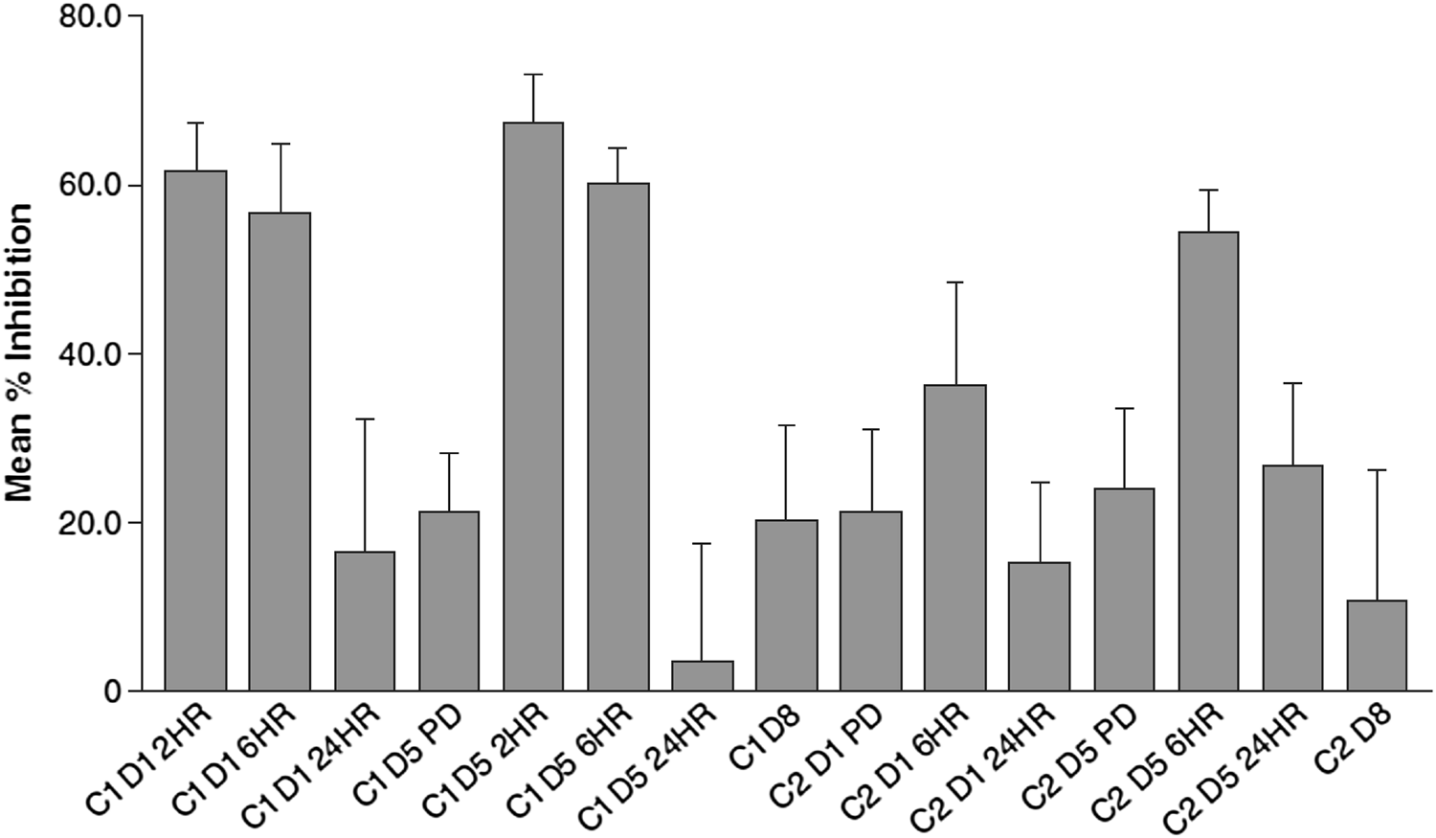
Inhibition of poly(ADP-ribose) polymerase (PARP) as measured by poly(ADP-ribose) (PAR) concentrations in peripheral blood mononuclear cells (PBMCs). The figure shows the PAR concentrations in PBMCs at study time points (*C* cycle, *D* day, *HR* hour, *PD* post-dose) expressed as a mean (SE) percentage inhibition from the pre-study baseline concentration

A PAR biomarker assay was performed to determine the degree of PARP inhibition in PBMCs as the dose of CEP-9722 was escalated in the study. The laboratory ELISA measures PAR in an extract from a processed cell pellet of PBMCs. As applied to the clinical setting, the assay was found to be sensitive to variations in sample handling and processing, which introduced considerable variability. However, a reduction in PAR concentrations relative to baseline was observed at the 2 and 6 h post-dose time points in cycle 1 when CEP-9722 was given without temozolomide. The reduction in PAR concentrations at the 6 h post-dose time point in cycle 2 was less marked (Fig. 2). There was no apparent relationship between dose of CEP-9722 and the degree of inhibition of PARP as measured by the PAR ELISA.

## Discussion

The exquisite sensitivity of tumor cells deficient in BRCA function to PARP inhibitors in vitro has generated much hope that this activity could be translated to clinical practice [18, 19]. Indeed, early clinical trials of monotherapy with the PARP inhibitor olaparib realized much of this promise with RECIST responses in 30–40 % of breast and ovarian cancer patients with BRCA mutations [14, 20]. Although a randomized phase 2 study of olaparib versus liposomal doxorubicin failed to show a benefit in progression-free survival in BRCA-mutated ovarian cancer patients [21], a randomized study of olaparib versus placebo as maintenance therapy following response to a platinum-based regimen did show such a benefit in patients with ovarian cancer [22]. This benefit in progression-free survival was statistically significant for both patients with and without BRCA mutations, indicating the broader potential for PARP inhibitors, and active clinical development for this indication is ongoing for many of the agents in the class [11].

The benefit of using a PARP inhibitor in combination with chemotherapy is less well established. An early study of rucaparib showed that this PARP inhibitor could be successfully combined with temozolomide [9]. In a subsequent phase 2 study of this combination in patients with metastatic melanoma, the progression-free survival was longer than that expected from historical controls; however, approximately half of the patients required a reduction in the dose of temozolomide due to enhanced myelosuppression [23]. Potentiation of the myelosuppressive effects of chemotherapy has been observed with other PARP inhibitors, including olaparib and veliparib, and this has limited the doses of chemotherapies that can be used in these combinations [24]. It was for this reason that CEP-9722 was of particular interest since nonclinical studies had shown that, at concentrations relevant to clinical use, CEP-8983 (the active form of CEP-9722) had only minor effects on the myelotoxicity of temozolomide [15].

In the current study, CEP-9722 was successfully combined with temozolomide at a dose of 150 mg/m^2^. Dose delays due to hematological toxicity were infrequent. Grade 3/4 neutropenia was not observed at any of the scheduled study visits. The MTD of CEP-9722 was eventually defined by asthenia and weight loss rather than hematological toxicity. Although this lack of potentiation of myelosuppression was encouraging, the degree of PARP inhibition achieved remains unclear. The PAR ELISA assay selected for the study had proven reliable in a laboratory setting, but was very sensitive to variations in sample handling and processing in the clinic. A clear reduction in PBMC PAR concentrations could be observed in samples taken 2 and 6 h post-dose, relative to a pre-dose baseline value; however, there was considerable variability and no apparent relationship to dose. The inhibitory effects on PAR production also were lost by the 24 h post-dose sample suggesting that a twice-daily dose might be a better treatment schedule.

The pharmacokinetics of CEP-8983 also showed a high degree of inter- and intra-patient variability. Notably, concentrations of CEP-8983 were lower in patients who were taking proton pump inhibitors and other medications that would raise gastric pH. This is likely to be a result of poorer absorption of CEP-8983, which is poorly soluble in nonacidic environments. Patients who smoked also had lower CEP-8983 plasma exposure, most probably due to increased metabolism by the smoking-induced increase in cytochrome CYP1A2.

Clinical activity in the form of RECIST response was limited to 1 patient in this study; a durable partial response was recorded in 1 patient treated at the highest dose of CEP-9722 (1,000 mg/day). Four additional patients (300–750 mg/day) had stable disease as best response. In view of the lack of potentiation of myelotoxicity of temozolomide, and the generally good tolerability observed, further clinical study of CEP-9722 would be warranted if the low and variable absorption of the drug could be overcome by an improved formulation. Twice-daily administration should also be considered.

## Conclusion

Data from previous preclinical and clinical studies have shown that PARP inhibitors potentiate the cytotoxicity of DNA-damaging chemotherapy and ionizing radiation [8]. The current study confirmed PARP inhibition by CEP-9722. CEP-9722 at a dose of 750 mg/day was the highest dose that was adequately tolerated in combination with temozolomide at a dose of 150 mg/m^2^. DLTs were nonhematological in nature. Pharmacokinetic and pharmacodynamic results indicate that further formulation development and a twice-daily dosing schedule should be considered.

## Electronic supplementary material

Below is the link to the electronic supplementary material.

Supplementary material 1 (DOCX 102 kb)
