# Supplementary figures and images for: Phase 1 dose-escalation study of the PARP inhibitor CEP-9722 as monotherapy or in combination with temozolomide in patients with solid tumors
Source: Cancer Chemother Pharmacol. 2014 Jun 1;74(2):257–65. doi: 10.1007/s00280-014-2486-9 (PMC4112042; doi:10.1007/s00280-014-2486-9)

**Supplemental Fig.** Dosing cycles (*C* cycle, *D* day, *QD* once daily, *TMZ* temozolomide)


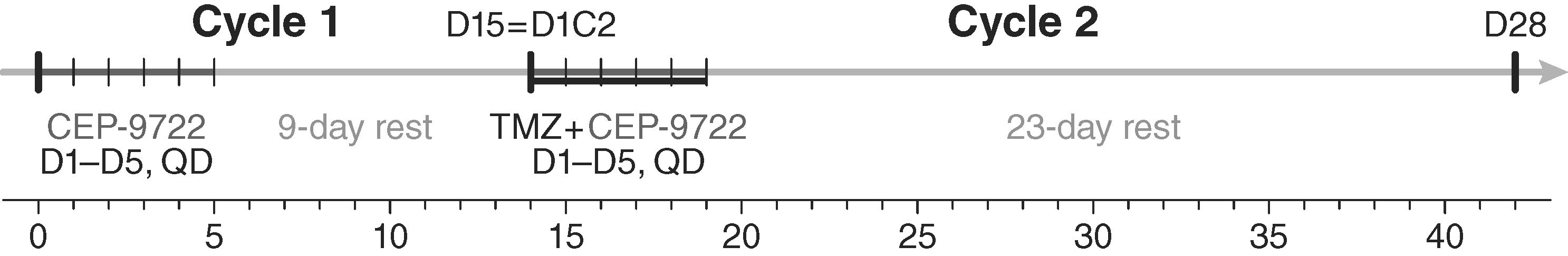

Supplement: Supplementary file 1 — Supplementary material 1 (DOCX 102 kb) [file 280_2014_2486_MOESM1_ESM.docx]
